# Regional versus General Anesthesia for Percutaneous Nephrolithotomy: A Meta-Analysis

**DOI:** 10.1371/journal.pone.0126587

**Published:** 2015-05-11

**Authors:** Henglong Hu, Baolong Qin, Deng He, Yuchao Lu, Zhenyu Zhao, Jiaqiao Zhang, Yufeng Wang, Shaogang Wang

**Affiliations:** Department and Institute of Urology, Tongji Hospital, Tongji Medical College, Huazhong University of Science and Technology, Wuhan, China; Taipei Medical University, TAIWAN

## Abstract

**Objective:**

To compare the effectiveness and safety of regional anesthesia (RA) and general anesthesia (GA) for percutaneous nephrolithotomy (PNL).

**Patients and Methods:**

PubMed, EMBASE, The Cochrane Library, and the Web of Knowledge databases were systematically searched to identify relevant studies. After literature screening and data extraction, a meta-analysis was performed using the RevMan 5.3 software.

**Results:**

Eight randomized controlled trials (RCTs) and six non-randomized controlled trials (nRCTs) involving 2270 patients were included. Patients receiving RA were associated with shorter operative time (−6.22 min; 95%CI, −9.70 to −2.75; p = 0.0005), lower visual analgesic score on the first and third postoperative day (WMD, −2.62; 95%CI, −3.04 to −2.19; p < 0.00001 WMD, −0.38; 95%CI, −0.58 to −0.18; p = 0.0002), less analgesic requirements (WMD, −59.40 mg; 95%CI, −78.39 to −40.40; p<0.00001), shorter hospitalization (WMD, −0.36d; 95%CI, −0.66 to −0.05; p = 0.02), less blood transfusion (RR, 0.61; 95%CI, 0.41 to 0.93; p = 0.02), fewer modified Clavion-Dindo Grade II (RR, 0.56; 95%CI, 0.37 to 0.83; p = 0.005), Grade III or above postoperative complications (RR, 0.51; 95%CI, 0.33 to 0.77; p = 0.001), and potential benefits of less fever (RR, 0.79; 95%CI, 0.61 to 1.02; p = 0.07), nausea or vomiting (RR, 0.54; 95%CI, 0.20 to 1.46; p = 0.23), whereas more intraoperative hypotension (RR, 3.13; 95%CI, 1.76 to 5.59; p = 0.0001) when compared with patients receiving GA. When nRCTs were excluded, most of the results were stable but the significant differences were no longer detectable in blood transfusion, Grade II and more severe complications. No significant difference in the total postoperative complications and stone-free rate were found.

**Conclusions:**

Current evidence suggests that both RA and GA can provide safe and effective anesthesia for PNL in carefully evaluated and selected patients. Each anesthesia technique has its own advantages but some aspects still remain unclear and need to be explored in future studies.

## Introduction

Percutaneous nephrolithotomy (PNL) has become the main treatment for large or multiple kidney stones, staghorn stones, and cases of failed shock wave lithotripsy. It also can be used to treat smaller sized stones with miniaturized instruments [1, 2]. PNL is usually performed under general anesthesia (GA) due to the better control of breathing and more comfort for the patients; all contraindications for GA apply to PNL according to the EAU guidelines on urolithiasis [1]. In the last two decades, several changes and modifications have taken place in an attempt to further refine the procedure and lower the morbidity, analgesic requirements, and duration of hospitalization. One of these changes has been the use of regional anesthesia (RA) including spinal anesthesia (SA), epidural anesthesia (EA), and combined spinal epidural anesthesia (CSEA) in patients who are undergoing PNL [3]. Some advantages of RA over GA had been shown in many surgeries [4–6], however, much of the effect of RA on PNL is still under veil. Since 2008, several studies have been carried out to compare the efficacy and safety of RA and GA [7–20]. As each type of anesthesia has some advantages and disadvantages and the results of such studies were not entirely consistent; a meta-analysis of the available evidence is needed to find their superiorities for PNL by comparing the outcomes of PNL under RA with those under GA. We hope the results would generate more interest in this topic and provide some help for urologists, anesthesiologists, patients, and policymakers in making relevant decisions in the future.

## Materials and Methods

### Literature search and study selection

A systematic literature search was performed using the PubMed, EMBASE, Web of Science databases, and The Cochrane Library to identify relevant studies following Cochrane standards and PRISMA (Preferred Reporting Items for Systematic Reviews and Meta-Analyses) guidelines [21]. The search was performed on July 20th, 2014 and updated on March 14th, 2015. The search was not limited by year or language. The initial search process was designed to find all published original articles involving the terms ("percutaneous nephrolithotomy" or "percutaneous nephrolithotripsy" or "PCNL" or "PNL") and ("anesthesi*" or "anaesthesi*" or "narco*"). Two authors independently screened all the citations and abstracts provided by this search strategy to identify potentially eligible studies. Only studies comparing RA and GA in PNL were included for further screening. Conference abstracts were not included because they were not deemed to be methodologically appropriate. Disagreements were resolved by discussion.

### Study quality assessment

The level of evidence was rated for each included study according to the criteria provided by the Oxford Center for Evidence-Based Medicine [22]. The methodological quality of the studies was assessed by two authors using the Newcastle-Ottawa Scale for nonrandomized controlled trials (nRCTs) and the Jadad scale for randomized controlled trials (RCTs) [23, 24]. Possible publication bias was assessed with funnel plots of the outcome comparisons.

### Data extraction and processing

Two reviewers reviewed the full texts of the included studies. Data including article name, author, published date and journal, patient number, age, gender, body mass index, American Society of Anesthesiologists (ASA) physical status, stone burden, stone location, anesthesia method and drugs, operative time, postoperative visual analgesic score (VAS), postoperative analgesic use, duration of hospitalization, intra-and postoperative complications, stone-free status, and follow-up method were extracted using a pre-designed data extraction form. The VAS measured at 1 hour following PNL was taken as the score of first postoprative day (POD) if it had been measured more than once within 24 hours after the operation. The reported postoperative complications were classified according to the modified Clavien-Dindo grading system [25]. To reduce the heterogeneity in analgesic requirements of different trials and make it easier to describe and understand, the equianalgesic dose was estimated using a morphine to tramadol equivalence ratio of 1:10 or a pethidine to tramadol equivalence ratio of 1:1 in studies that used morphine or pethidine instead of tramadol to control postoperative pain [26, 27]. Any discrepancies between the extracted data were resolved by discussion.

### Statistical analysis

A meta-analysis was performed to compare the safety and efficacy of RA in PNL with GA using Review Manager Software (RevMan v.5.3, Cochrane Collaboration, Oxford, UK). Dichotomous data were presented as relative risk (RR) and continuous outcomes were presented as weighted mean difference (WMD), both with 95% confidence interval (CI). For studies presenting continuous data as means and range, standard deviations were calculated using the methodology described by Hozo and colleagues [28]. The chi-square test and I^2^ statistics were used to evaluate the heterogeneity among studies. Moreover, the pooled estimates were calculated with the fixed-effect model if no significant heterogeneity was detected; otherwise, the random-effect model was used. The pooled effects were determined by the z test. A p-value less than 0.05 was considered statistically significant. Funnel plots were generated using RevMan v.5.3. Additionally, a sensitivity analysis was performed by pooling only RCTs, if nRCTs were included in the outcome comparison.

## Results

### Description of included studies and quality assessment

**Fig 1:**
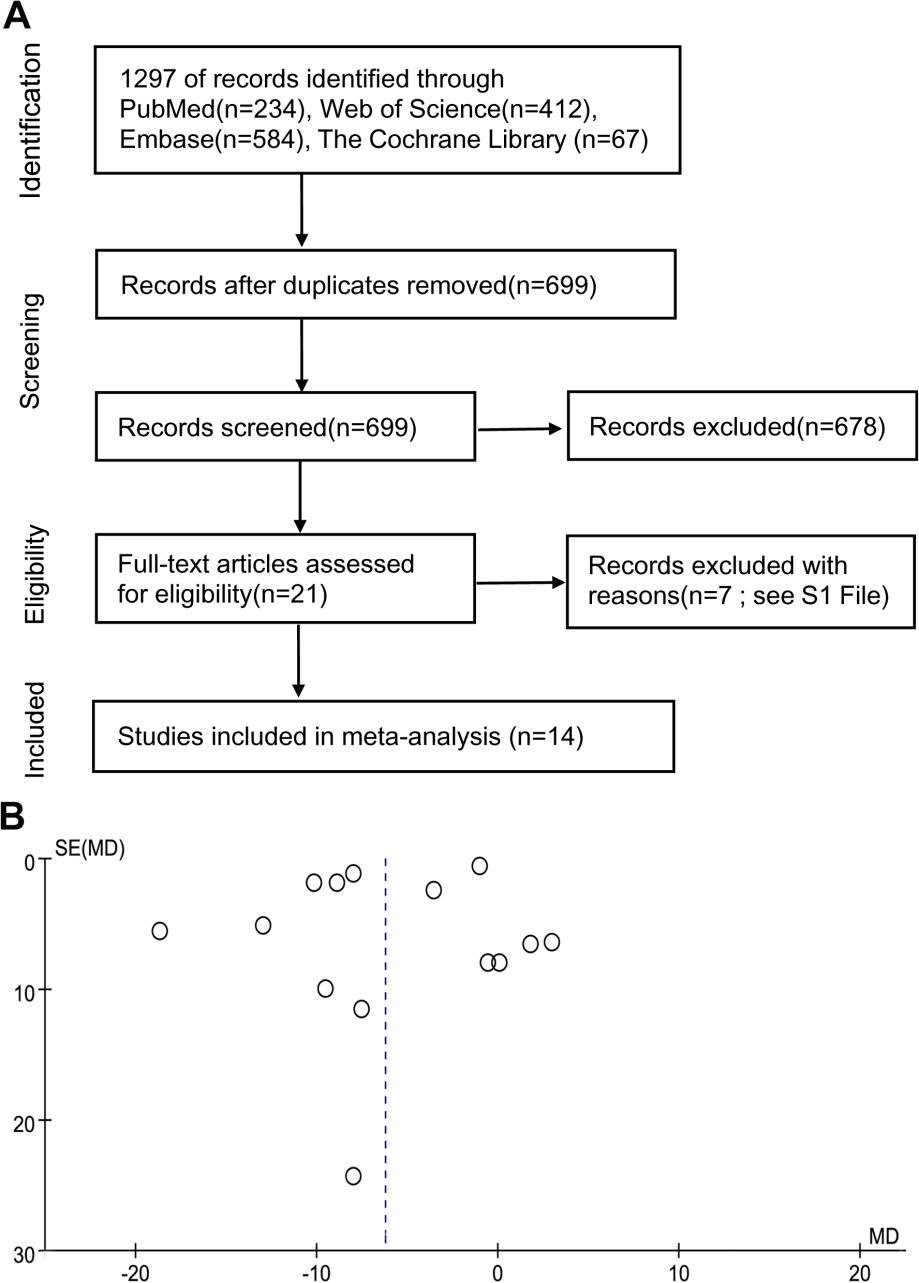
(A) Flowchart of the studies selection process. (B) Funnel plot of operative time.

Fourteen studies were selected for the analysis, including 1095 patients who underwent PNL under RA and 1175 under GA. Fig 1A shows the literature search and study selection process. Table 1 presents the basic characteristics of each study included in our meta-analysis. The reported patient baseline characteristics such as age, sex ratio, body mass index, ASA status, stone burden, and location were comparable in each study according to the article. Some studies also reported other baseline characteristics that may have an effect on the outcomes we studied: degrees of hydronephrosis [10, 17], the calyx punctured (lower/ middle/ upper) [9, 20], subcostal and supracostal punctures [8–10, 15, 18, 19], the punctured access number [12, 18, 19], whether a double J stent was inserted [9, 12, 19], and whether a nephrostomy tube after the surgery was placed [9–12, 14–17, 19]. All these data were also comparable between the two groups in those studies. Some literatures also presented the size of nephrotomy tube [9,14,16,17] and nephroscope [9–11,16–19] which is same in the two groups, but the size of nephrotomy in one study [15] is various and they did not analyze the data for any difference between the groups. Only the comparison of operative time included all the 14 studies, and the funnel plot did not show obvious asymmetry, in other words, there may be no publication bias (Fig 1B). The other 14 funnel plots are presented in S1 Fig Most of them seemed symmetrical except for the funnel plots of postoperative analgesic demand (S1F Fig), nausea and vomiting (S1H Fig), and Grade II postoperative complications (S1L Fig). It is inappropriate to assess whether these funnel plots were truly symmetrical or not using statistical tests because they should be used only when there are at least 10 studies included. If asymmetry does exist, the former two’s asymmetry may be explained by heterogeneity, publication bias or chance. The asymmetry of the last one may result from publication bias or chance [29].

**Table 1:** Characteristics and methodological quality of included studies.

Study	Year	Study design	Level of evidence	Study quality	Anesthesia technique	Sample size	Age(year) (M±SD)	Sex (M/F)	BMI	ASA score	Stone area or maximal diameter
Elbealy [7]	2008	RCT	Level 2	3/5	RA(EA)	19*	41±13	19/0	28.1	Ⅰ15 Ⅱ5 Ⅲ0	ND
					GA	20	39±15	20/0	27.4	Ⅰ15 Ⅱ5 Ⅲ0	ND
Karacalar [8]	2009	RCT	Level 2	3/5	RA(CSEA)	86	48.64±16	45/41	27.6±7.2	Ⅰ35 Ⅱ51 Ⅲ0	1137.78mm^2^
					GA	90	48.32±15	47/43	27.8±6.2	Ⅰ37 Ⅱ53 Ⅲ0	598.4mm^2^
Singh [9]	2011	RCT	Level 2	3/5	RA(CSEA)	32	40.06±13.41	22/10	ND	ND	2195.31±643mm^2^
					GA	32	39.66±13.7	18/14	ND	ND	2271±620mm^2^
Tangpaitoon [10]	2012	RCT	Level 2	3/5	RA(EA)	24	53.04±13.53	17/7	21.25±3.21	Ⅰ10 Ⅱ13 Ⅲ1	40.8 ± 16.4mm
					GA	26	56.69±11.32	16/10	21.36±3.98	Ⅰ7 Ⅱ18 Ⅲ1	35.4 ± 15.0mm
Mehrabi [11]	2013	RCT	Level 2	3/5	RA(SA)	58	47.4±7.6	31/27	24.1±7.2	all<Ⅲ	32.8 ± 9.6mm
					GA	52	43.7±8.2	35/17	24.1±5.6	all<Ⅲ	30.9 ± 10.6mm
Nouralizadeh [12]	2013	RCT	Level 2	3/5	RA(SA)	50	41.16±11.2	29/21	ND	ND	55.1±28.7mm
					GA	50	42.66±13.61	27/23	ND	ND	55.6±29.5mm
Movasseghi [13]	2014	RCT	Level 2	3/5	RA(SA)	29	39.6±9.7	19/10	26.4±3.8	Ⅰ23 Ⅱ6 Ⅲ0	ND
					GA	30	46.9±13.6	19/11	28.1±4.6	Ⅰ22 Ⅱ8 Ⅲ0	ND
Moawad [14]	2015	RCT	Level 2	2/5	RA(SA)	100	44±11	60/40	27.1±4.1	Ⅰ65 Ⅱ35 Ⅲ0	31.9±7.4mm
					GA	100	43±11	64/36	27.4±2.1	Ⅰ69 Ⅱ31 Ⅲ0	33.7±6.3mm
Kuzgunbay [15]	2009	CS	Level 3	8/9	RA(CSEA)	37	44±15	24/13	ND	ND	731±394mm^2^
					GA	45	45±15	26/19	ND	ND	734±386mm^2^
Kim [16]	2013	CS	Level 3	9/9	RA(CSEA)	77	54.8±12.2	47/30	25.1±3.9	ND	34.5±24.0mm
					GA	24	50.8±17.8	14/10	23.3±2.8	ND	42.3±36.1mm
Moslemi [17]	2013	CS	Level 3	8/9	RA(SA/EA)	54	39	ND	25	ⅠandⅡ59 Ⅲ5	ND
					GA	69	41	ND	26	ⅠandⅡ55**Ⅲ4	ND
Cicek [18]	2014	CS	Level 3	8/9	RA(SA)	440	48.8±14.03	283/157	ND	Ⅰ238Ⅱ169Ⅲ33	533.9±480.94mm^2^
					GA	564	47.2±13.83	344/220	ND	Ⅰ305Ⅱ218Ⅲ39	529.5±324.12mm^2^
Gonen [19]	2014	CS	Level 3	9/9	RA(SA)	29	45.6±13.6	18/8	ND	ND	558.6±297.2mm^2^
					GA	20	40.8±12.9	13/7	ND	ND	630.7±486.2mm^2^
Karatag [20]	2015	CS	Level 3	7/9	RA(SA)	63	45.8±14.6	ND	27.0±4.9	ND	155.08±84.9mm^2^
					GA	53	30.3±22.1	ND	25.8±7.1	ND	151.00±75.5mm^2^

BMI: body mass index ASA: American Society of Anesthesiologists RCT: randomized controlled trial RA: regional anesthesia EA: epidural anesthesia ND: not demonstrated GA: general anesthesia CSEA: combined spinal epidural anesthesia SA: spinal anesthesia CS: cohort study

* Originally twenty patients included in the RA group, but one patient converted to GA.

**Totally 69 patients were included in the GA group, the authors [16] might report a wrong number here.

### Operative time

**Fig 2:**
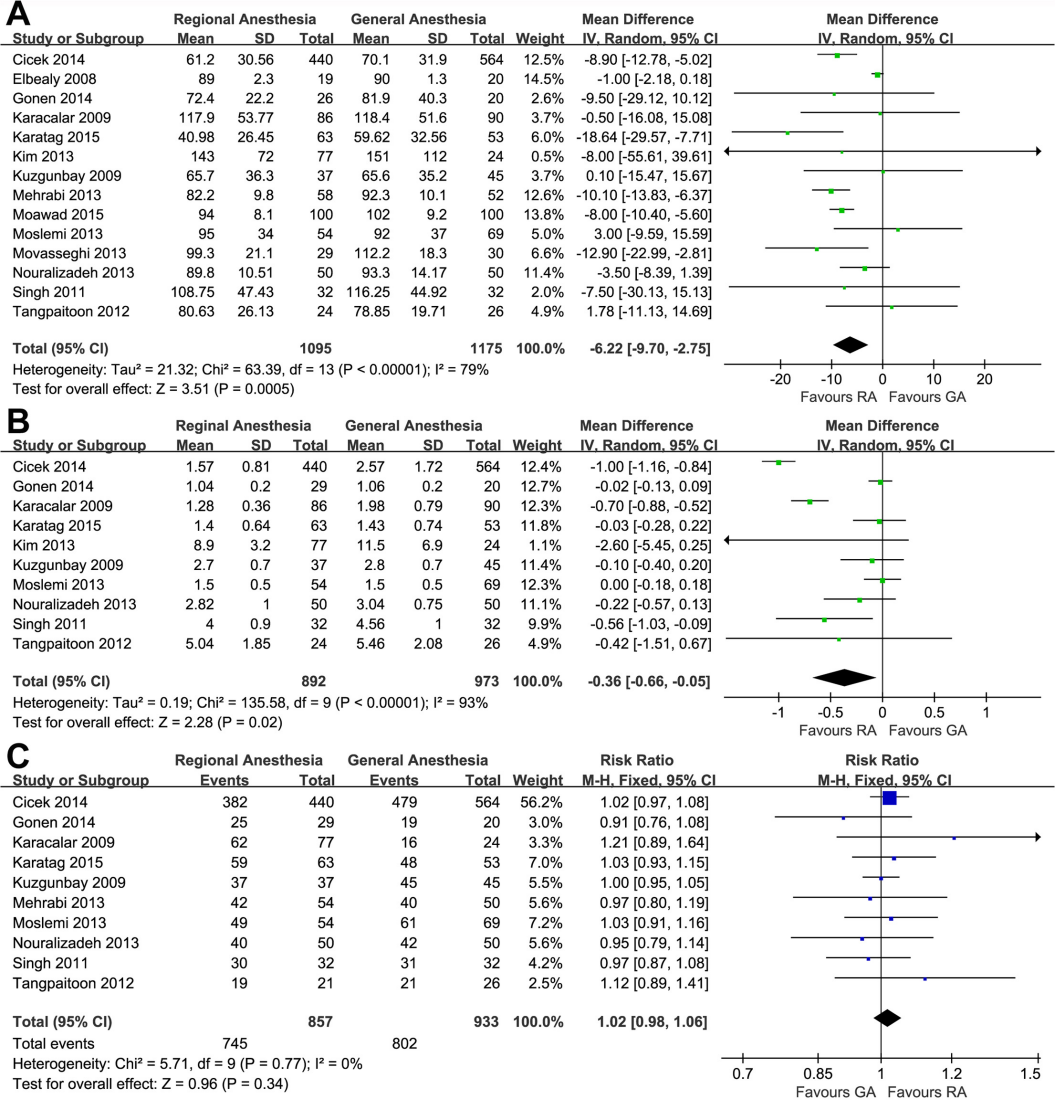
Forest plots and meta-analyses of (A) operative time, (B) hospital stay and (C) stone-free rate.

Meta-analysis of 14 studies by a random effects model demonstrated that the operative time of RA group was 6.22 minutes shorter than that of the GA group (95%CI, −9.70 to −2.75; p = 0.0005; Fig 2A).

### Hospital stay

Meta-analysis of 10 studies in a total of 1865 patients showed that the RA group was discharged 0.36 d earlier than the GA group (95%CI, −0.66 to −0.05; p = 0.02; Fig 2B).

### Stone-free status

Pooling the data from 10 studies that assessed the postoperative stone-free status revealed no significant difference between the RA and GA groups (RR, 1.02; 95%CI, 0.98 to 1.06; p = 0.34; Fig 2C)

### VAS and analgesic requirement

**Fig 3:**
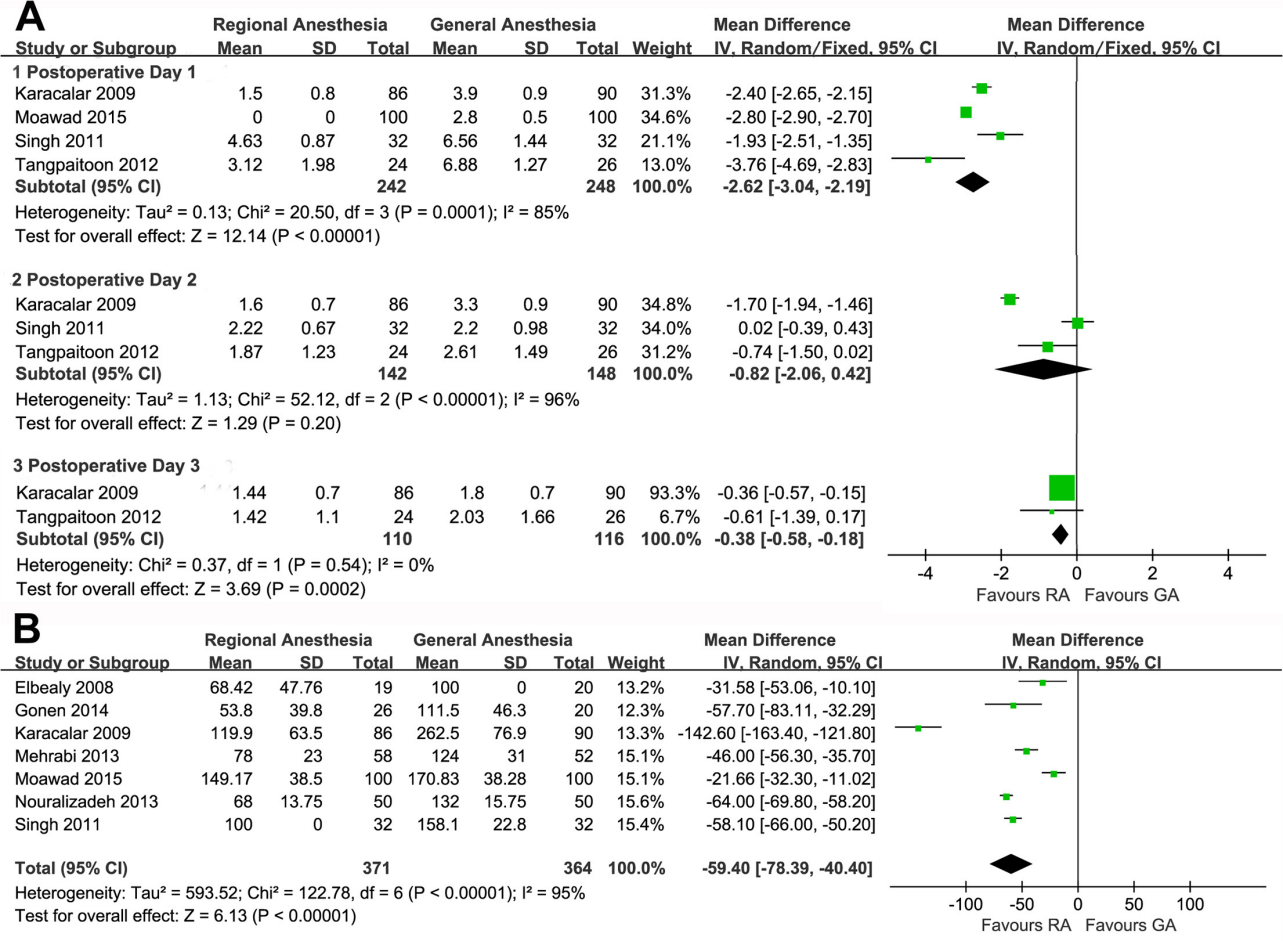
Forest plots and meta-analyses of (A) VAS on the 1^st^, 2^nd^ and 3^rd^ postoperative day, and (B) analgesic requirement.

Six studies assessed postoperative pain using VAS but two of them provided incomplete data. The meta-analysis of available data showed that RA was associated with less pain on POD 1 (WMD, −2.62; 95%CI, −3.04 to −2.19; p<0.00001; Fig 2A) and POD 3 (WMD, −0.38; 95%CI, −0.58 to −0.18; p = 0.0002; Fig 2A). However, we found a similar trend with no statistical significance on POD 2 (WMD, −0.82; 95%CI, −2.06 to 0.42; p = 0.20; Fig 3A).

Though 12 studies provided postoperative analgesic data, only 7 with explicit analgesic dose met the analysis requirements. After the equianalgesic conversion, a meta-analysis was carried out which showed that the patients receiving RA demanded less tramadol to relieve postoperative pain (WMD, −59.40mg; 95%CI, −78.39 to −40.40; p<0.00001; Fig 3B) and this was consistent with the VAS comparison.

### Perioperative complications

**Fig 4:**
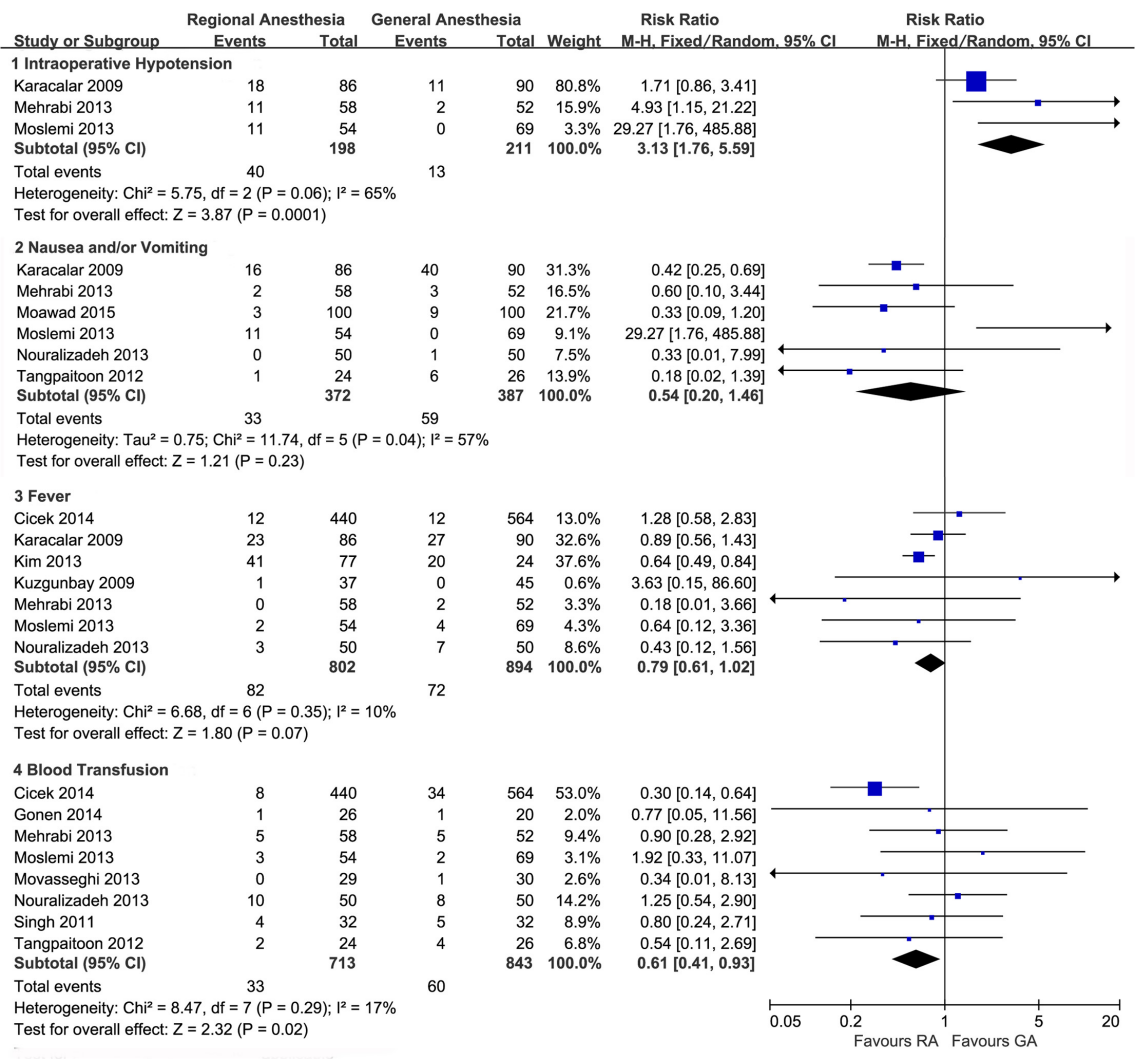
Forest plots and meta-analyses of intraoperative hypotension, nausea and/or vomiting, fever and blood transfusion.

All the studies reported all or some aspects of the intra- and/or postoperative complications. Patients who underwent PNL in RA, rather than in GA, were associated with a higher risk of intraoperative hypotension (RR, 3.13; 95%CI 1.76 to 5.59; p = 0.0001; Fig 4), blood transfusion (RR, 0.61; 95%CI, 0.41 to 0.93; p = 0.02; Fig 4), and a potential lower risk of postoperative fever (RR, 0.79; 95%CI, 0.61 to 1.02; p = 0.07; Fig 4), whereas the nausea and/or vomiting risk showed no significant difference between the two groups (RR, 0.68; 95%CI, 0.17 to 2.68; p = 0.58; Fig 4).

**Fig 5:**
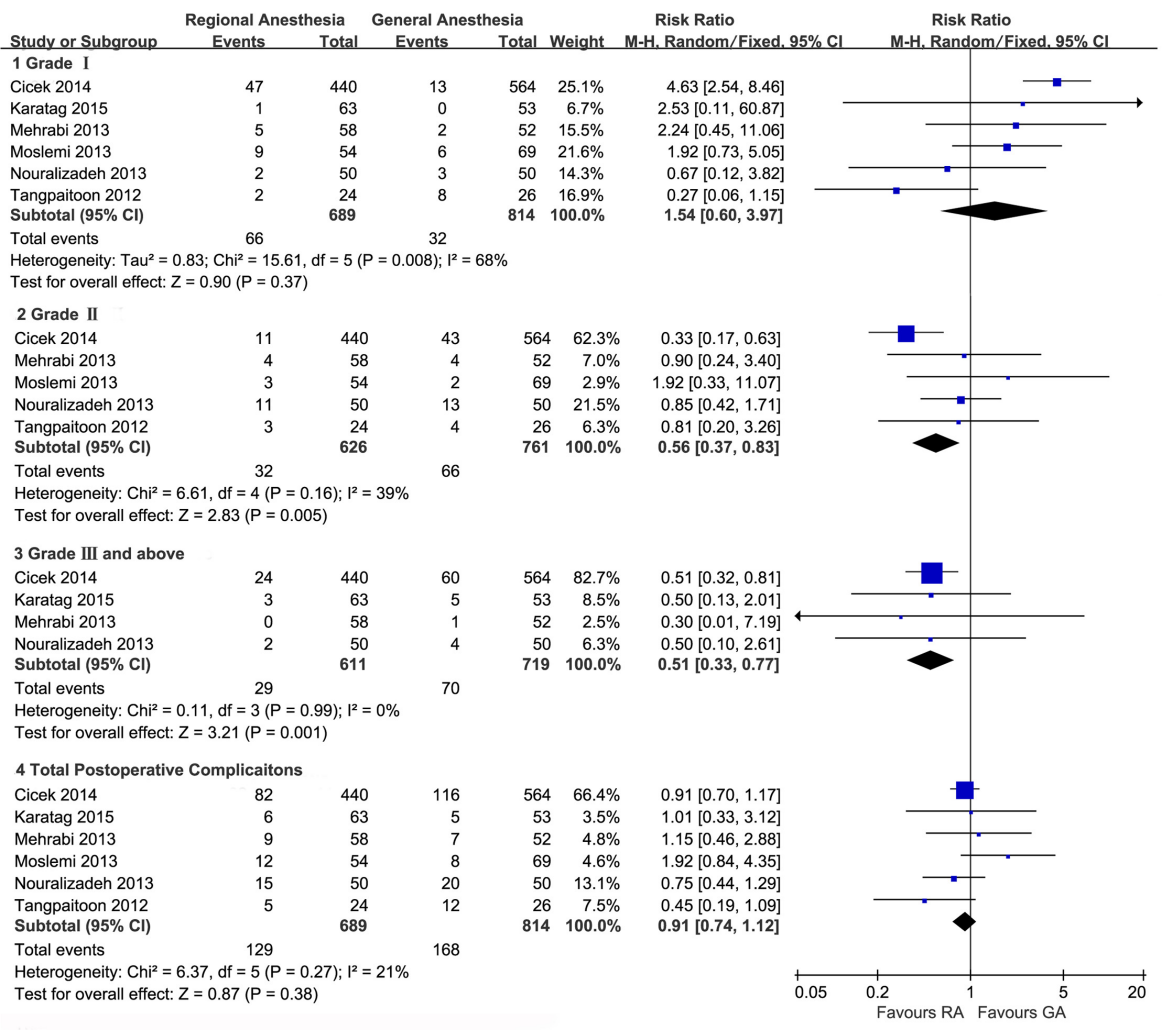
Forest plot and meta-analyses of postoperative complications.

Pooling the data from 6 studies that assessed the postoperative complications revealed that the total postoperative complications was not associated with the type of anesthesia (RR, 0.91; 95%CI, 0.74 to 1.12; p = 0.38; Fig 5). However, compared to patients receiving GA, those receiving RA seemed to experience more Grade I complications (RR, 1.54; 95%CI, 0.60 to 3.97; p = 0.37; Fig 5) though without statistical significance, fewer Grade II (RR, 0.56; 95%CI, 0.37 to 0.83; p = 0.005; Fig 5), and fewer Grade III or more severe complications (RR, 0.51; 95%CI, 0.33 to 0.77; p = 0.001; Fig 5).

### Sensitivity analysis

The sensitivity analysis suggested that the results of this meta-analysis were relatively stable (Table 2). When the nRCTs were excluded, most of the outcomes including postoperative time, stone-free rate, hospital stay, analgesic demand, intraoperative hypotension, postoperative fever, and total postoperative complications were not greatly affected. Nevertheless, the reduced sample number failed to find significant differences of blood transfusion, and Grade II and more severe postoperative complications between the two groups. Though these significant differences were no longer detectable in the sensitivity analysis, the preference remained the same. One exception was the Grade I postoperative complications, which were found to occur less in the RA group, ac finding contradicting the overall analysis results; however, this difference still remained insignificant. Unexpectedly, a significant association was found between RA and reduced risk of nausea and/or vomiting in the sensitivity analysis (RR, 0.39; 95%CI, 0.25 to 0.60; p<0.0001; Table 2).

**Table 2:** Sensitivity analysis results.

Items	Studies	Sample size	Tests for heterogeneity		Analysis model	Test for overall effect		RR/WMD	Favors
		RA/GA	I^2^	P*		Z	P*	95%CI	
Operative Time(min)	[7–14]	398/400	85%	<0.00001	Random	2.63	0.009	-5.55 [-9.68,-1.41]	RA
Tramadol demand(mg)	[7–9,11,12,14]	345/344	96%	<0.00001	Random	5.64	<0.00001	-59.68 [-80.41,-38.94]	RA
Complications									
IOH	[8,11]	144/142	41%	0.19	Fixed	2.56	0.01	2.24 [1.21,4.15]	GA
Nausea/Vomiting	[8,10–12,14]	318/318	0%	0.92	Fixed	4.23	<0.0001	0.39 [0.25,0.60]	RA
Fever	[8,11,12]	194/192	5%	0.35	Fixed	1.30	0.19	0.75 [0.48,1.16]	RA
Blood Transfusion	[9–13]	193/190	0%	0.85	Fixed	0.36	0.72**	0.90 [0.52,1.56]	RA
Grade I POC	[10–12]	132/128	46%	0.16	Fixed	0.88	0.38	0.69 [0.30,1.58]	RA
Grade II POC	[10–12]	132/128	0%	0.99	Fixed	0.56	0.58**	0.85 [0.48,1.50]	RA
≥Grade III POC	[11,12]	108/102	0%	0.78	Fixed	1.09	0.27**	0.44 [0.10,1.91]	RA
Total POC	[10–12]	132/128	5%	0.35	Fixed	1.45	0.15	0.74 [0.49,1.11]	RA
Hospital Stay(d)	[8–10,12]	192/198	49%	0.12	Fixed	7.73	<0.00001	-0.59[-0.74,-0.44]	RA
Stone-Free Rate	[8–12]	234/182	0%	0.48	Fixed	0.42	0.68	1.02 [0.93,1.12]	RA

RA: regional anesthesia GA: general anesthesia RR: relative risk WMD: weighted mean difference CI: confidence interval IOH: intraoperative hypotension POC: postoperative complications

* P<0.05 was considered statistically significant.

**originally significant before nRCTs excluded.

## Discussion

Anesthesia is definitely important for surgeries and the common choice of anesthetic technique mainly depends on patient and surgeon preferences, feasibility of technique in a given patient, intra-and postoperative pain control, skills of anesthesiologist, and perioperative costs [7].

Last three decades have witnessed a large amount of studies on the efficacy and safety of PNL, however, the effect of anesthesia-type on this surgery was rarely discussed by urologists and anesthesiologists. PNL is routinely performed under GA due to better control of breathing and it being more comfortable for the patients. However there are some occasional side effects from GA such as pulmonary complications, drug allergy, and postoperative nausea and vomiting [10]. In addition, tracheal tube displacement and neurologic events especially at the time of shifting position from lithotomy to prone are important concerns during PNL under GA [12]. Besides those, two inherent and indisputable facts about RA are its relatively low costs, which is about four times cheaper [11], and good patient cooperation during anesthesia. The latter provides great advantages in preventing pulmonary and neurologic events during position shifting and supracostal puncture as patients can follow verbal commands and control respiration. All the above facilitated the trying of a series of local and regional anesthesia methods such as interpleural block, renal capsular block, peritubual infiltration, EA, SA, and CSEA in PNL [30–33].

Earlier studies focusing on PNL with RA were only small case series of selected patients with low evidence grade. Since 2008, several comparative studies including RCTs on this problem have been carried out, but some of the results are controversial. We systematically reviewed the outcomes of PNL under RA and GA, and performed a meta-analysis of 14 studies (2270 patients). We observed no significant difference between the two groups in terms of baseline characteristics and this laid the foundation for reasonable comparisons.

Operative time of PNL mainly depends on patient characteristics, surgeon’s experience, and anesthesia. The definition of operative time was not clearly stated in all but four studies which calculated it as the time elapsed from the puncture of the pelvicalyceal system until placement of the nephrostomy tube [9, 15] or the duration between the insertion of the ureteral catheter and the removal of the microperc system from the kidney [14, 20]. The operative time was significantly longer in the GA group and Sugiharan et al reported that longer operative time was associated with a higher risk of severe complications after PNL [34].

Another outcome in favor of RA was the lower postoperative VAS, with a difference reaching statistical significance on POD 1 and POD 3. All three studies reported less VAS in RA on POD 2 while the detected WMD of −0.82 did not reach statistical significance. This may be due to the continuation of pain relief provided by RA into the postoperative period. Accordingly, the postoperative analgesic demand was significantly lesser in the RA group, which may reduce the risk of analgesic side effects. Patients probably will get better quality of life and sooner recovery if the postoperative pain is lesser.

Different complications favor different anesthesia types. Intraoperative hypotension seemed to occur more in the RA group, which could be effectively managed with ephedrine. In contrast, the frequency of other complications including blood transfusion, nausea and vomiting, and fever in RA group were lower than in the GA group. The reduced blood transfusion may be caused by the lower thoracic pressure during surgery, decreased blood pressure and vasodilation following sympathetic block and shorter operative time [18, 35]. Major complications (Grade II and above) were seen significantly more frequently in group GA than in RA, but no such trend was observed for minor complications. They occurred more frequently in the RA group in the overall analysis but an opposite conclusion could be drawn when nRCTs were excluded. However, the number of RCTs was only three (260 patients) and the differences were still insignificant. Further studies are needed to answer this question. No significant difference was detected in the total postoperative complications in all trials. After the sensitivity analyses were carried out, some of the outcome differences turned out to be insignificant, but the trends did not change. Thus, it can be concluded that PNL under RA is quite safe, maybe even safer than GA considering the significantly lesser major complications. It should also be pointed out that cardiac arrest was reported to happen under EA during PNL [36], but none of the patients experienced the same in these included trials. Therefore, a patient who is to undergo PNL must be evaluated well before he or she can receive RA as the anesthesia method.

In addition, RA yielded significantly shorter hospitalization duration, which may be due to lesser pain and major complications. This also could be regarded as a cost-saving feature of RA.

Overall, we found no statistically significant difference in the stone-free rate. Another important aspect of assessing treatment outcome is patient and doctor-satisfaction. Two studies [10, 11] assessed patient satisfaction after anesthesia and surgery and one study [8] assessed surgeon satisfaction. The use of different scales to assess the degree of satisfaction made it difficult to perform a meta-analysis. Of the three studies, two [8, 10] demonstrated patients receiving RA as having higher level of satisfaction than the GA group, while the other showed no significant difference between the two groups. This may result from lesser pain and complications. In addition, the surgeon satisfaction was similar in the two groups.

Beside the aspects discussed above, there are at least two important aspects that need to be further investigated. One of them is the fluoroscopy time. In nearly all the studies, PNL was performed in the prone position under fluoroscopic guidance. Cicek and colleagues reported that the fluoroscopy time was significantly shorter in RA groups (4.56 ± 2.8 vs. 5.06 ± 2.83 min) [18], however, another study found it was not affected by the method of anesthesia [14]. The other aspect needing further investigation is the post anesthesia recovery. Karacalar et al. [8] found that the time spent in post anesthesia care unit was significantly shorter in the RA group than in the GA group (4.9 ± 8.7 min vs.18.6 ± 4.2 min) according to the standardized discharge criteria, however, Movasseghi and colleagues [13] found no difference between the two groups.

From the above, we can find some merits of RA over GA, but RA does have some demerits. For instance, there are many contraindications to RA, such as a patient’s inability to maintain stillness during the needle puncture, raised intracranial pressure, and skin or soft tissue infection at the proposed site of needle insertion. Another one is that it is unbearable for patients under RA to be in prone position if the operative time is too long. Moreover, the patient’s anxiety can be a major deterrent in maintaining the respiratory cycle—hyperventilation by an anxious patient can be a major problem during PNL puncture. Last but not the least, if the patient has to be switched to GA, or open surgery in case of an emergency, it might take a great amount of time to reposition the patient and administer GA. None but 5 patients converted to open surgery in the 2270 patients encountered a failed RA. These are a reminder to evaluate well a patient who is to undergo PNL before he or she can receive RA as the anesthesia method.

There are several limitations to be considered in interpreting the results of our meta-analysis. First, similar to other studies comparing RA and GA in surgeries [4, 6], a relatively heterogeneous inclusion criteria was used. Three RA techniques (SA, EA, and CSEA) instead of one specific technique were compared with GA. They all belong to intrathecal anesthesia and the differences among them could be unremarkable when comparing with GA. Second, most comparisons included nRCTs. Though sensitivity analysis showed that most of the results were stable and the tendency remained unchanged, the difference of blood transfusion and of Grade II and more severe complications between the two groups became statistically insignificant when the nRCTs were removed. More RCTs are needed to verify these indefinite outcomes. Third, our meta-analysis only included studies published in indexed journals. We did not search for unpublished studies or original data. The small number of included studies in all the comparisons except for the operative time limited the ability to detect publication bias, although the funnel plot of operative time results seems to suggest that there was no evidence of publication bias. Finally, all the patients in the studies were adults without morbid obesity and diseases contraindicating RA, for example, some type of back deformity. Additionally, all the studies excluded patients whose ASA scores were more than III. In children and patients with ASA IV and V or other special conditions, these outcomes should be interpreted cautiously.

## Conclusion

In conclusion, the present meta-analysis shows that both RA and GA can provide safe and effective anesthesia for PNL in carefully evaluated and selected patients. Each anesthesia technique has its own advantages and RA is associated with shorter operative time, less postoperative pain, less analgesic requirements and faster hospital discharge, whereas higher intraoperative hypotension. But some aspects still remain unclear and need to be explored in future studies.

## Supporting Information

S1 Fig The funnel plots of comparisons. Funnel plots of (A) hospital stay, (B) stone-free status, visual analgesic score of the first (C), second (D), third (E) postoperative day, (F) postoperative analgesic demand, (G) intraoperative hypotension, (H) nausea and vomiting, (I) postoperative fever, (J) blood transfusion, (K)Grade I, (L) Grade II, (M) Grade III or more sever and (N) total postoperative complications.
(TIF)

S1 File List of full-text excluded articles with reasons for exclusion. (PDF)

S2 File PRISMA checklist. (DOC)
