# Supplementary figures and images for: Regional versus General Anesthesia for Percutaneous Nephrolithotomy: A Meta-Analysis
Source: PLoS One. 2015 May 11;10(5):e0126587. doi: 10.1371/journal.pone.0126587 (PMC4427359; doi:10.1371/journal.pone.0126587)

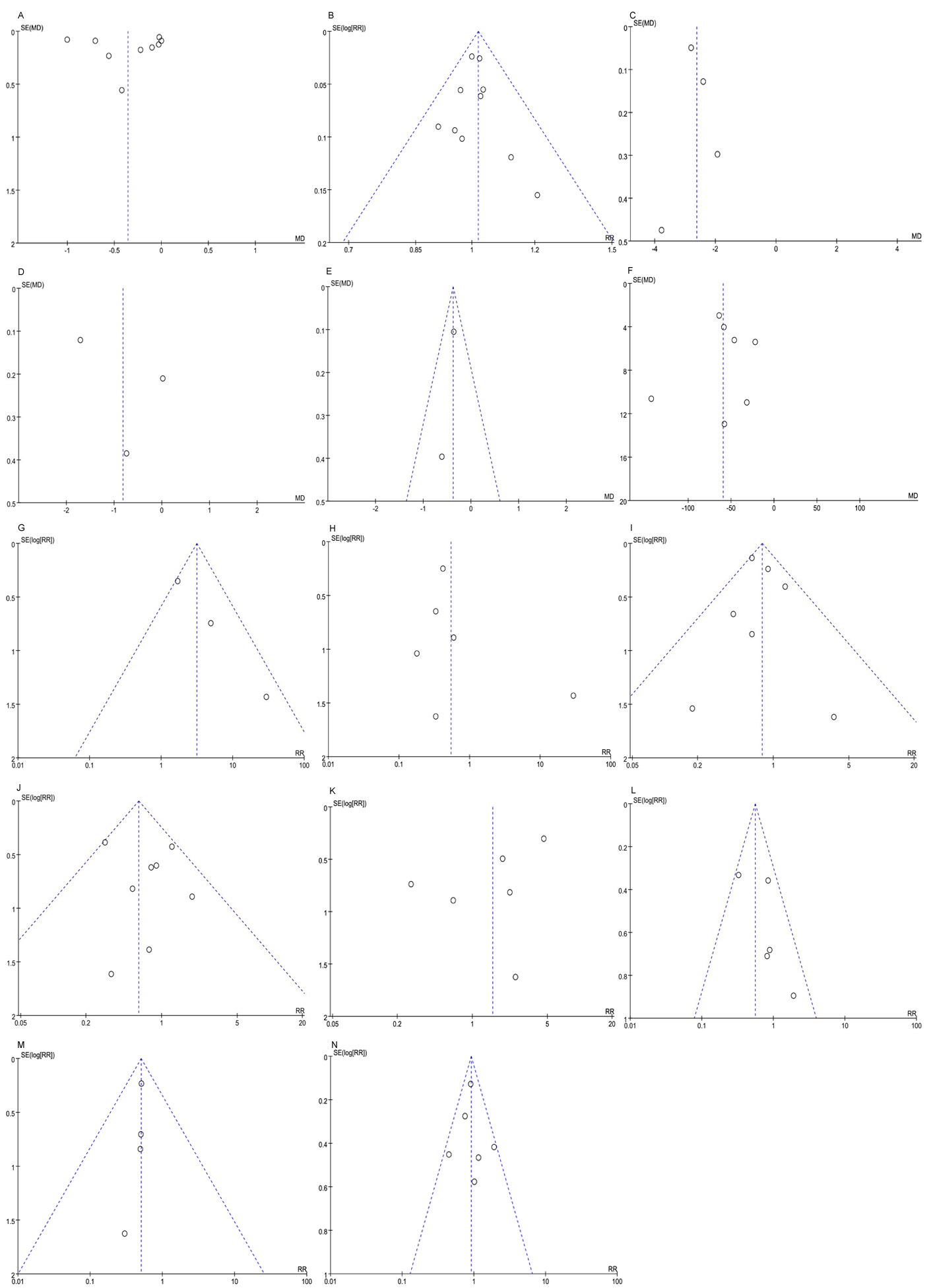

Supplement: S1 Fig — Funnel plots of (A) hospital stay, (B) stone-free status, visual analgesic score of the first (C), second (D), third (E) postoperative day, (F) postoperative analgesic demand, (G) intraoperative hypotension, (H) nausea and vomiting, (I) postoperative fever, (J) blood transfusion, (K)Grade I, (L) Grade II, (M) Grade III or more sever and (N) total postoperative complications. (TIF) [file pone.0126587.s001.tif]
