# Supplementary material for: Regional versus General Anesthesia for Percutaneous Nephrolithotomy: A Meta-Analysis
Source: PLoS One. 2015 May 11;10(5):e0126587. doi: 10.1371/journal.pone.0126587 (PMC4427359; doi:10.1371/journal.pone.0126587)
Supplement: S1 File — (PDF) [file pone.0126587.s002.pdf]

The list of full-text excluded articles with reasons for exclusion

1. Sharifi SHH, Soltani MH, Rezaeetalab GH, Sharif RY, Khaledi F, et al. Intermittent Perirenal Instillation of Bupivacaine After Tubeless Percutaneous Nephrolithotomy Under Spinal Anesthesia: A Double-Blind, Placebo-Controlled Clinical Trial. Journal of Endourology. 2014;28:1299-1303.

Reason for exclusion: There was no general anesthesia control group in this study and the trial was not focus on comparing general anesthesia with regional anesthesia.

2. Mehrabi S, Shirazi KK. Results and Complications of Spinal Anesthesia in Percutaneous Nephrolithotomy. Urology Journal. 2010;7:22-25.

Reason for exclusion: There was no general anesthesia control group in this study.

3. El-Husseiny T, Moraitis K, Maan Z, Papatsoris A, Saunders P, et al. Percutaneous Endourologic Procedures in High-Risk Patients in the Lateral Decubitus Position Under Regional Anesthesia. Journal of Endourology. 2009;23:1603-1606.

Reason for exclusion: Besides percutaneous nephrolithotomy, other percutaneous endourologic procedures were also investigated in this study. The data was mixed and was not available for this meta-analysis.

4. Wu SD, Yilmaz M, Tamul PC, Meeks JJ, Nadler RB. Awake Endotracheal Intubation and Prone Patient Self-Positioning: Anesthetic and Positioning Considerations During Percutaneous Nephrolithotomy in Obese Patients. Journal of Endourology. 2009;23:1599-1602.

Reason for exclusion: This authors reviewed the literature regarding anesthetic and positioning considerations in obese patients and also describe their own technique. No original data was available for this meta-analysis.

5. Qiming HAN. Combined spinal-epidural anesthesia and Propofol for percutaneous nephrolithotomy. China Journal of Modern Medicine. 2009;19:880-882.

Reason for exclusion: There was no general anesthesia control group in this study and the trial was not focus on comparing general anesthesia with regional anesthesia.

6. Rozentsveig V, Neutander AZ, Roussabrov E, Schwartz A, Lismer L, et al. Anesthetic considerations during percutaneous nephrolithotomy. Journal of Clinical Anesthesia. 2007;19:351-355.

Reason for exclusion: There was no regional anesthesia control group in this study and the trial was not focus on comparing general anesthesia with regional anesthesia.

7. Nadler RB, Monk TG, Elashry O, Nakada SY, McDougall EM, et al. Simultaneous bilateral percutaneous nephrolithotomy with subarachnoid spinal anesthesia. Journal of Endourology. 1998;12:27-31.

Reason for exclusion: There was no general anesthesia control group in this study and the trial was not focus on comparing general anesthesia with regional anesthesia.
